# Research on Envelope Profile of Lithium Niobate on Insulator Stepped-Mode Spot Size Converter

**DOI:** 10.3390/mi16010109

**Published:** 2025-01-19

**Authors:** Jianfeng Bao, Dengcai Yang, Zhiyu Chen, Jingyuan Zhang, Feng Yang

**Affiliations:** 1 School of Physics and Optoelectronic Engineering, Institute of Laser Engineering, Beijing University of Technology, Beijing 100124, China; baojf@emails.bjut.edu.cn (J.B.); dengcaiyang@bjut.edu.cn (D.Y.); 17764531229@163.com (J.Z.); 2 Microwave Photonics Technology Laboratory, Chengdu 610036, China; chenzhiyu_1986@163.com

**Keywords:** LNOI, coupling, SSC, stepped, envelope

## Abstract

To enhance the end-face coupling efficiency of lithium niobate on insulator (LNOI) chips, in conjunction with current device fabrication processes, a stepped spot size converter (SSC) based on a special outer envelope profile has been proposed and investigated. This stepped SSC can reduce the coupling loss between the LNOI waveguide and a normal single-mode optical fiber. First, the output waveguide of a mode converter was proposed and simulated, in which the mode field had the biggest overlapping integral factor with a single-mode fiber (MDF ≈ 9.8 μm). Then, a stepped LNOI waveguide, the basic structure of the mode converter, with three kinds of outer envelope profile, was proposed and analyzed. Through analysis of the impacts of different envelope profiles on mode spot conversion efficiency, the relationship between envelope profile and propagation efficiency was obtained. Additionally, the rule of LNOI stair height variation tendency and the pattern of mode spot conversion efficiency for the multi-step mode spot converter in LNOI were obtained. Ultimately, a stepped SSC with a COS-shaped envelope curve was adopted. When this stepped SSC is coupled to single-mode fiber with a mode-field diameter of 9.8 μm, the coupling efficiency of the TE mode was 95.35% at the wavelength of 1550 nm.

## 1. Introduction

Optical waves have emerged as the primary medium for conveying information with increasing amounts of information, which means that optical waveguide devices have become a research hotspot. Photoelectric integrated chips, which boast advantages such as low cost, low power consumption, and high performance, and are capable of integrating various active and passive photoelectric devices into a single unit, and have become the focus of much research. The external connections of optical chips are typically established through optical fiber coupling to facilitate information transfer. The common coupling methods between optical fibers and on-chip waveguides include grating coupling [1,2,3] and end-face coupling [4,5,6]. Grating coupling, owing to its structural characteristics, exhibits wavelength selectivity, in which the coupling fiber and waveguide are not coaxial, posing challenges in packaging and making it commonly used in on-chip testing. In contrast, end-face coupling is characterized by low coupling loss, wide operational bandwidth, and ease of packaging. To facilitate low-loss packaging applications, end-face coupling is often adopted as the primary means for coupling and outputting optical signals on-chip, thereby possessing broad application prospects and demand.

Currently, most studies on optical chips are based on thin film materials. However, when thin films are used as waveguide materials, the large refractive index difference between the waveguide core layer and the cladding layer results in a significant difference in the modal field distribution between the waveguide and the optical fiber. Direct coupling between them often leads to significant modal field mismatch, which is an important cause of loss in end-face coupling. Therefore, modal field reshaping is required to improve coupling efficiency. For the optical fiber, tapered fibers [7] can be employed to reduce the diameter of the emitted light spot. For the chip, the coupling structure can be designed to enlarge the mode spot at the end face [8]. On the silicon-on-insulator (SOI) chip platform, various coupling structures have been proposed and implemented, such as three-dimensional enlargement of the waveguide output port [9], utilizing inverted tapered waveguides and cladding to achieve mode-field expansion [10,11,12,13], and using subwavelength gratings to reduce the refractive index of the waveguide material [14,15,16].

Lithium niobate (LN) possesses exceptional electro-optic, acousto-optic, and nonlinear properties, making it an excellent material for the fabrication of optical devices [17]. Bulk LN can be processed into high-speed electro-optic modulators through proton exchange or diffusion techniques, which are widely used in high-speed communications and microwave photonics. With the emergence and development of LN thin film materials [18,19,20], research on LN thin film-based waveguides has flourished, leading to the development of various devices, such as intensity modulators [21], phase modulators [22], microring resonators [23], racetrack resonators [24], polarization rotators [25], and optical filters [26]. Due to the high refractive index contrast of LN thin film waveguides, the cross-sectional dimensions of the waveguides are smaller, resulting in stronger light confinement. This allows for the creation of smaller waveguide devices and integrated multifunctional devices based on LN thin films, making it an excellent platform for monolithic integration and an ideal solution for optoelectronic chip-integrated systems.

Although the high refractive index contrast of LN thin films enhances the confinement of light in waveguides, it also reduces the mode-field diameter of the waveguides to less than 1 μm^2^. The direct alignment and coupling of optical fibers to these waveguides would result in significant coupling loss due to mode-field mismatch. Therefore, mode spot converters are designed to enhance their coupling efficiency with optical fibers. The structures of LN mode spot converters primarily include single-layer inverted taper structures, multilayer inverted taper structures, and multilayer step structures [27,28,29,30,31,32,33,34,35,36]. Single-layer inverted taper mode spot converters gradually narrow the width of the LN waveguide, reducing the equivalent refractive index at the waveguide cross-section in the horizontal direction [27,28]. This weakens the confinement of light in the LN waveguide, allowing light to propagate into specially designed cladding, thereby achieving mode spot conversion and reducing coupling loss. Multilayer inverted taper structures further enhance the coupling efficiency between optical fibers and waveguides [29,30,31,32,33,34] by reducing the waveguide dimensions in both the horizontal and vertical directions, further weakening the confinement of light in these directions and significantly reducing coupling loss. Multilayer step structures are proposed because of the small size at the tip of inverted taper structures, which typically require high-precision alignment using electron beam lithography (EBL) for photolithography. However, EBL is not suitable for full-wafer processing. Therefore, researchers have proposed using i-line contact exposure with sequential overlay lithography and etching to form multilayer step structures, reducing the waveguide height in the vertical direction to decrease the equivalent refractive index of the waveguide and thus expand the mode field [35,36]. However, there is still a research gap regarding how to set the length and height of the steps in step-mode spot converters to achieve higher-quality coupling.

In this paper, the structure of an LNOI stepped-mode spot converter is investigated. The light transmission efficiency of the mode spot converter is analyzed via the equivalent refractive index method and the beam propagation method. Simulation comparisons are conducted to evaluate the coupling efficiency of mode spot converters with typical step envelope profiles such as *y* = cos*x*, *y* = a*x*, *y* = *x*^2^, y = *x*^3^, $y=\sqrt{x}$ and $y=\sqrt[{3}]{x}$. The influence of various parameters in the coupling structure on the coupling efficiency is analyzed, and an optimized design approach is proposed. Ultimately, an LNOI stepped-mode spot converter with an outer envelope profile shaped such as y = cosx was obtained. The simulations revealed that the TE mode spot conversion efficiency of this structure at a wavelength of 1550 nm is as high as 97.1%, and the maximum mode coupling efficiency can reach 95.35%, while coupling with a single-mode fiber with a mode field diameter of 9.8 μm.

## 2. Methodology

**Figure 1:**
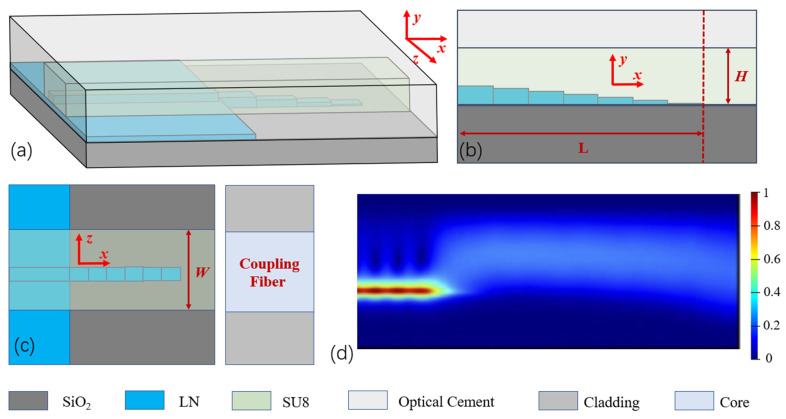
Structure of the step-mode spot converter: (**a**) three-dimensional schematic, (**b**) schematic of the transmission cross-section, (**c**) top view of the waveguide, and (**d**) schematic of the transmitted optical field.

The LNOI step-mode spot converter designed in this paper, as shown in Figure 1a,b, is a structure that exhibits a stepped distribution in the vertical direction of the waveguide near its end face. The transmission of light in the LNOI step mode spot converter and the optical fiber is illustrated in Figure 1c,d. Along the light transmission direction, due to the gradual decrease in the height of the waveguide steps, the equivalent mode refractive index decreases, resulting in a weaker confinement of light by the LNOI waveguide. This allows light near the edge of the mode spot converter to gradually enter the SU8 cladding waveguide, which has a smaller refractive index and a cross-sectional mode-field distribution close to that of the optical fiber, thereby completing the mode spot conversion. Finally, the light is transmitted from the SU8 waveguide into the optical fiber, achieving light coupling from the waveguide to the fiber.

The process of end-face coupling can be divided into two stages: the mode-field matching stage at the end face, and the mode conversion stage inside the chip. During the mode-field matching stage at the chip end face, the light emitted from the optical fiber is incident on the chip end face, and some energy is dissipated into the surrounding air due to reflection and scattering. The vertical incidence reflectivity (*R*) and transmittance (*T*) at the dielectric interface can be obtained using Fresnel’s laws [37]. The physical quantities used in the mathematical model are shown in Table 1.

(1) $$R=\frac{{\left(n_{1}-n_{2}\right)}^{2}}{{\left(n_{1}+n_{2}\right)}^{2}},$$

(2) T=1−R,

where *n*_1_ and *n*_2_ represent the refractive indices of the media on both sides of the interface, respectively. Obviously, the reflectivity (*R*) increases as the difference in refractive indices between the media on both sides of the interface increases, resulting in a decrease in the optical power transmitted into the chip. Only the light that is mode-matched upon transmission into the chip can successfully enter the waveguide for propagation. The matching ratio [37] between the two modes can be expressed by the mode-field overlap integral factor (*η*) as follows:

(3) $$\eta =\frac{{\left|\int E_{1}\cdot E_{2}\cdot dA\right|}^{2}}{\int {\left|E_{1}\right|}^{2}\cdot dA\cdot \int {\left|E_{2}\right|}^{2}\cdot dA},$$

where *E*_1_ and *E*_2_ represent the electric field intensities of the two modes, respectively, and *dA* denotes an infinitesimal area element. Owing to the difference in size between the optical fiber and the transmission waveguide, there is a significant disparity in the spot sizes at the two ends, resulting in a relatively low mode-field overlap integral factor (*η*). This is the primary cause of coupling loss between different end faces.

With respect to the mode conversion process, each step waveguide in the step mode spot converter has the same equivalent refractive index for its respective level, wheresa steps of different heights possess different equivalent refractive indices. Consequently, the transmission of light through the step-mode spot converter can be equivalently considered as light passing through a cascade of waveguides with progressively changing refractive indices. Therefore, the total transmittance (*T_all_*) of the mode spot converter can be expressed as follows:

(4) $$T_{all}=a_{1}\left(1-R_{1}\right)a_{2}\left(1-R_{2}\right)\cdot \cdot \cdot a_{n-1}\left(1-R_{n-1}\right)=a_{all}T_{1}T_{2}\cdot \cdot \cdot T_{n-1},$$

where *T_all_*, *a_all_*, and *n* are the total transmittance, the total coupling coefficient of all parts, and the nth interface, respectively. *R_n_* is the reflectivity between level n and level n + 1, *T_n_* is the transmittance between level n and level n + 1, and *a_n_* is the mode coupling coefficient between level n and level n + 1. *T_all_* increases with increasing *T_n_* and *a_all_* but decreases with increasing *n*. Combining Equations (1), (2), and (4), it can be concluded that reducing the equivalent refractive index difference between chip interfaces and decreasing the number of interfaces can lower coupling losses. For step-structured mode spot converters, the equivalent refractive indices at their input and output end faces can be considered fixed values, with each step having the same fixed equivalent refractive index. There exists an equivalent refractive index difference between adjacent steps of different heights. When light transmitted from one step to another, it can be regarded as crossing an interface. Increasing the number of steps can reduce the refractive index difference, but an increase in the number of steps will also increase coupling losses. Therefore, it is necessary to reasonably design the number of steps and the trend in equivalent refractive index changes, i.e., to design an appropriate step envelope profile at the structural level, in order to improve transmittance and achieve efficient mode spot conversion.

To investigate the impact of several profile shapes on the coupling performance of LNOI step-mode spot converters, this paper employs the Finite Difference Eigenmode (FDE) and Finite Difference Time Domain (FDTD) methods to simulate the processes of end-face coupling and mode spot conversion. We design a cladding structure for the mode spot converter that is compatible with the mode field of a conventional single-mode fiber, and explore the relationship between the mode spot conversion efficiency and the coupling efficiency of LNOI step-mode spot converters with typical profile envelopes such as y = cosx, y = ax, y = x^2^, y = x^3^, $y=\sqrt{x}$, and $y=\sqrt[{3}]{x}$ and stepped envelopes.

## 3. Simulation and Analysis

The degree of mode-field matching is determined by the overlap integral of the mode fields between the optical fiber and the end face of the mode spot converter. Therefore, to achieve good matching between the mode spot converter and the optical fiber mode field, the FDE and FDTD methods were used to calculate and simulate the mode evolution and transmission from the optical fiber to the cladding section of the mode spot converter in order to determine the cladding parameters of the mode spot converter and the refractive index of the medium in the coupling region. Based on the properties of LN, the single-mode transmission conditions for LNOI optical waveguides can be obtained. As the mode converter is primarily used for coupling between the waveguide on the LNOI multifunctional chip and the external optical fiber, the electro-optic modulator, which is the core device on the LNOI chip, can function properly only when the fundamental TE mode light is transmitted. Therefore, all the studies in this paper focused solely on the fundamental TE mode. Combined with the stepped structure mentioned in this paper, the following parameters were set: the LN thin film thickness h_tf_ = 400 nm, insulating layer (SiO_2_) thickness = 4.7 μm, the thin film orientation X-cut, a mode spot converter cladding of SU8 resin, and the outermost medium of the cladding being an optical adhesive.

**Figure 2:**
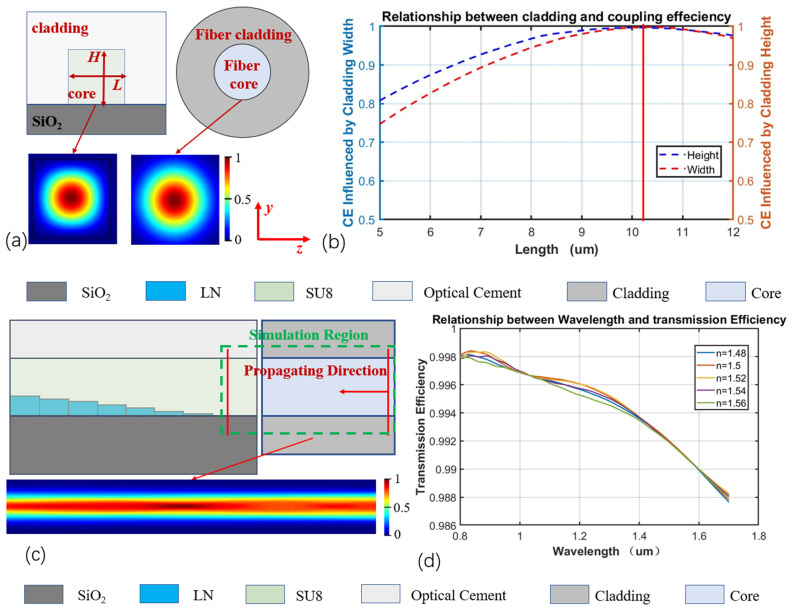
(**a**) Sectional mode-field diagram, (**b**) relationship between cladding width and thickness and integration factor of optical fiber mode-field overlap, (**c**) coupling model between mode-spot converter and optical fiber, and (**d**) relationship between wavelength and transmission efficiency.

First, to determine the cladding parameters of the mode spot converter shown in Figure 1, end-face models of the stepped-mode spot converter and the optical fiber were constructed via the FDE method. The simulated cross-sectional mode field is shown in Figure 2a, where W and H represent the width and thickness of the SU8 cladding, respectively. By scanning the width W and height H, a relationship curve between the cladding width and thickness and the overlap integral factor of the optical fiber’s mode field were obtained, as shown in Figure 2b. When the SU8 cladding width W and height H of the mode spot converter are both 10.2 μm, the overlap integral factor between the optical fiber and the mode spot converter’s end-face mode field is the highest, reaching 98.2%. Therefore, the cladding width W and height H of the mode spot converter are determined to be 10.2 μm. Under the condition of maximizing the mode-field overlap integral factor, to achieve efficient coupling between the optical fiber and the mode spot converter, a refractive index matching medium is added at their coupling gap to enhance the coupling efficiency throughout the coupling process. An FDTD-based coupling model of the mode spot converter and optical fiber is established as shown in Figure 2c, and the optical coupling efficiency of different refractive index matching media within the wavelength range of 800–1600 nm is simulated, as shown in Figure 2d. It can be observed that within the range of 800–1600 nm, the mode spot converter based on this structure and the optical fiber exhibit an extremely wide optical bandwidth and coupling efficiency. When the refractive index of the matching medium n_gap_ is 1.52, the coupling efficiency between the optical fiber and the cladding of the mode spot within the simulated spectral range reaches its highest value and varies most smoothly. After achieving mode-field matching between the waveguide end-face and the optical fiber end face, i.e., determining the equivalent refractive index of the mode spot converter’s end face, it is necessary to reasonably design the stepped structure so that the entire mode spot converter and optical fiber coupling achieve the best coupling effect. At this point, the simulation of the coupling process between the optical fiber and the mode spot converter is complete, and the cladding parameters of the mode spot converter are determined: the SU8 cladding width W and height H are both 10.2 μm, and the refractive index of the matching medium n_gap_ is 1.52.

For the analysis of the mode spot conversion process, the Finite Element Method (FEM) employed to calculate the equivalent refractive index of each step in the step-structured mode spot converter and the optical transmission efficiency of the converter. The relationship between the envelope profile and the transmission efficiency of the mode spot converter is analyzed. Subsequently, the FDTD method will be used to model and simulate the aforementioned process, calculating the transmission characteristics of the LNOI step-structured mode spot converter enveloped by curved profiles.

**Figure 3:**
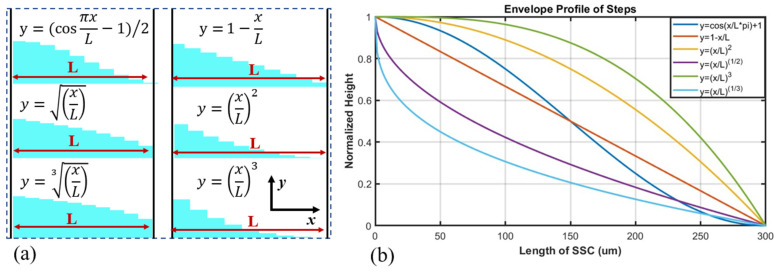
(**a**) Schematic diagram of the step structure, (**b**) step envelope curves.

A three-dimensional schematic diagram of the mode spot converter with a step structure based on special profiles is shown in Figure 1a. The LN waveguide near the output end face consists of LN step waveguides enveloped by a specific profile, with the cladding material being SU8 resin, which is encapsulated by an optical adhesive (Norland 65) with a certain refractive index. A schematic diagram of the optical transmission cross-section in the step-structured mode spot converter is shown in Figure 1b. The step cross-section can be designed with a special profile, and the figure illustrates the y = cos x profile. The top view and end-face view of the coupling relationship between the mode spot converter and the optical fiber are shown in Figure 1c and Figure 2a, respectively. The fiber core diameter is close to the width and height of the cladding of the mode spot converter, with the coupling end-face core being SU8 and the cladding being optical adhesive (Norland 65). The step envelope profiles studied and analyzed are shown in Figure 3a, with all profiles having the same starting and ending points.

The step envelope profiles are illustrated in Figure 3b, where the selected profiles can be classified into four categories. The first category is the linear profile represented by $y=1-\frac{x}{L}$, which signifies a uniform change in step height. The second category includes profiles with step heights that decrease rapidly first and then slowly, represented by curves with powers greater than 1, including $y={\left(\frac{x}{L}\right)}^{2}$ and $y={\left(\frac{x}{L}\right)}^{3}$. The third category is profiles that are symmetrical about the linear profile *y* = 1 − *x*/L, with step heights that decrease slowly first and then rapidly, represented by curves with powers less than 1, which contain $y=\sqrt{\left(\frac{x}{L}\right)}$ and $y=\sqrt[{3}]{\left(\frac{x}{L}\right)}$. The fourth category is trigonometric function curves, where the initial and final slopes are zero, and the step heights decrease slowly first, then rapidly, and finally slowly again, represented by the curve $y=(\cos\frac{\pi x}{L}-1)/2$.

**Figure 4:**
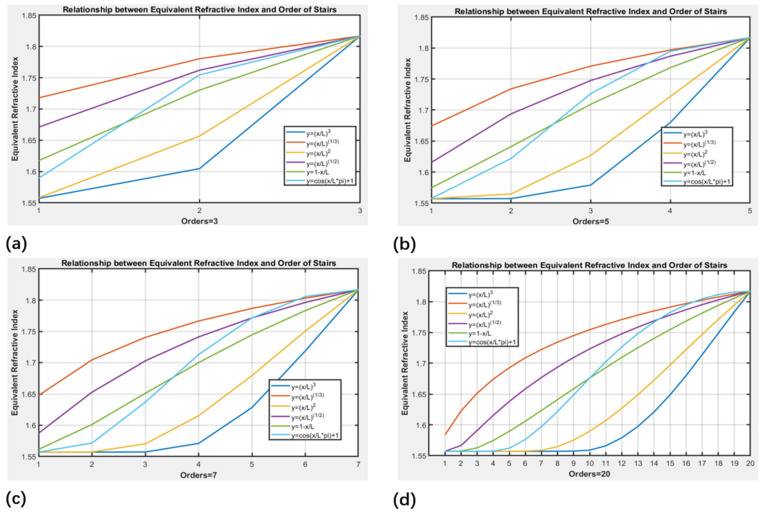
Equivalent refractive index of the transmission cross-section of stepped SSC with 5 kinds of profile envelopes under different orders of stairs: (**a**) order = 3, (**b**) order = 5, (**c**) order = 7, and (**d**) order = 20.

For the calculation of the optical equivalent refractive index, we constructed a 2D model of the stepped SSC transmission cross-section via FEM tools from COMSOL, which are based on Maxwell’s equations. The height, width, and refractive index parameters of the stepped transmission cross-section model constitute the boundary conditions within the simulation area. The calculation boundaries employed perfect matching layer (PML) boundary conditions to simulate the external conditions of the waveguide light guiding structures. By using the FEM, which is based on the step height and width parameters, the equivalent refractive index of the transmission cross-section of step-structured mode spot converters with steps enveloped by different profiles can be obtained, as shown in Figure 4. Figure 4a,b,c and d, respectively, exhibit the equivalent refractive indices corresponding to the cross-sections of each step in the mode spot converters with a total of 3, 5, 7, and 20 steps. It can be observed that the distribution curves of the equivalent mode refractive indices follow the same trend as the step envelope curves. However, when the number of steps is small, for profiles with powers less than 1, such as $y=\sqrt{\left(\frac{x}{L}\right)}$ and $y=\sqrt[{3}]{\left(\frac{x}{L}\right)}$, the heights of the first few steps are greater, resulting in larger equivalent refractive indices. The initial equivalent refractive indices of the other profiles are closer to those of the SU8 cladding, implying smaller refractive index differences and larger transmission losses.

**Figure 5:**
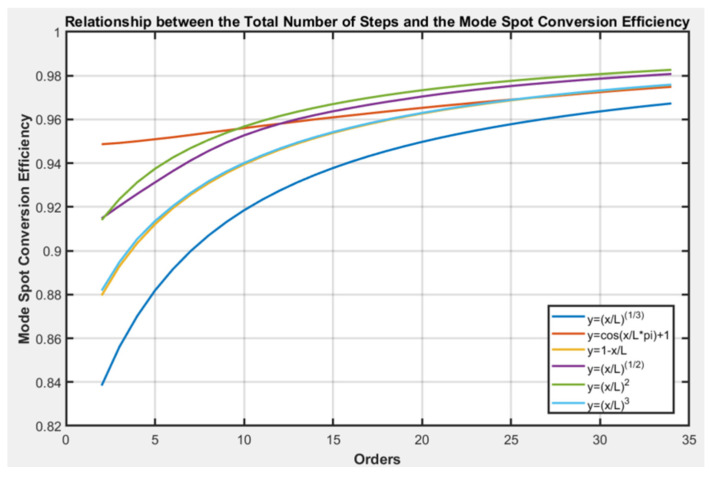
Relationship between the total number of steps and the mode spot conversion efficiency.

Using the FDTD method, the relationship between the number of stepped envelopes of different line types and the total coupling coefficient can be calculated. On the basis of the comprehensive equations for steps (1), (2), and (4), the relationship between the total number of steps and the mode spot conversion efficiency can be derived, as shown in Figure 5. It can be observed that for the simulated curves, the overall mode spot conversion efficiency tends to stabilize as the number of steps increases, indicating that a greater total number of steps leads to a higher step mode spot conversion efficiency. When the number of steps is less than 10, there are significant differences in the conversion efficiencies of different envelope mode spots, with the highest efficiency for the y = (cos(πx/L−1))/2 envelope, followed by the $y={\left(\frac{x}{L}\right)}^{2}$, $y=\sqrt{\left(\frac{x}{L}\right)}$, $y={\left(\frac{x}{L}\right)}^{3}$, $y=1-\frac{x}{L}$ and $y=\sqrt[{3}]{\left(\frac{x}{L}\right)}$. When the number of steps exceeds 10, the mode spot conversion efficiency is highest for the $y={\left(\frac{x}{L}\right)}^{2}$ linear envelope. Except for the $y=(\cos\frac{\pi x}{L}-1)/2$ linear envelope, the trends in mode spot conversion efficiency with the number of steps are similar for the other linear envelope types. It can be concluded that the $y=(\cos\frac{\pi x}{L}-1)/2$ linear envelope maintains a higher mode spot conversion efficiency when the number of steps is relatively small.

**Figure 6:**
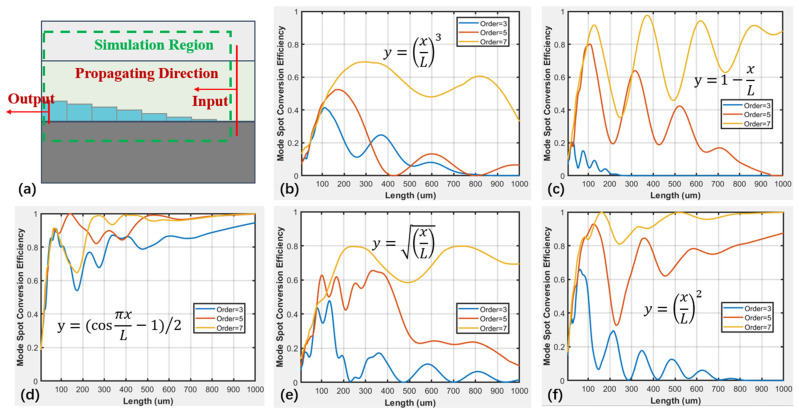
Simulation diagram of transmission efficiency of different envelope step spot converters. (**a**) Schematic diagram of simulation model, (**b**) $y={\left(\frac{x}{L}\right)}^{3}$ line, (**c**) $y=1-\frac{x}{L}$ line, (**d**) $y=(\cos\frac{\pi x}{L}-1)/2$ line, (**e**) $y=\sqrt{\left(\frac{x}{L}\right)}$ line, and (**f**) $y={\left(\frac{x}{L}\right)}^{2}$ line.

**Figure 7:**
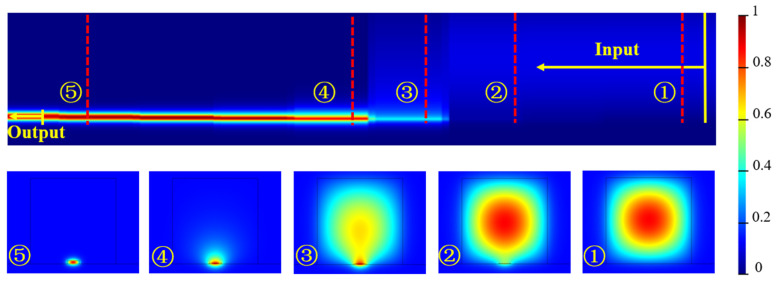
$y=(\cos\frac{\pi x}{L}-1)/2$. Schematic diagram of linear light field transmission.

To validate the mode spot conversion efficiency of step mode spot converters with different linear envelope types when the number of steps is relatively small, a model as shown in Figure 6a was established via the FDTD method. FDTD from Lumerical/ANSYS was used to simulate the light transmission process in the stepped SSC. The calculation boundaries are shown in Figure 6a. PML boundary conditions were used to simulate the external conditions. Finally, the relationship between the conversion efficiency of the stepped SSC in different linearly shaped envelopes and the total length of the steps, and as shown in Figure 6b–f, can be seen. By setting envelopes with different linear types and scanning the length of the mode spot converter with 3, 5, and 7 steps, the relationships between transmission efficiency and linear types and orders of steps is presented. As the length of the mode spot converter increases, there is a certain oscillation in the mode spot conversion efficiency. When the length of the mode spot converter is less than 200 μm, the mode spot conversion efficiency of several linear types increases with the increasing number of steps. As shown in Figure 6d, the step mode spot converter based on the $y=(\cos\frac{\pi x}{L}-1)/2$ linear type has the largest and least fluctuating mode spot conversion efficiency. The transmission field distribution of light in the stepped SSC based on the $y=(\cos\frac{\pi x}{L}-1)/2$ linear type is shown in Figure 7. The optical signal is gradually drawn into the LN waveguide from the SU8 layer as the LN stair increases after the light enters the SSC. When the number of steps is 5 and the length of the mode spot converter is 395 μm, the highest mode spot conversion efficiency reaches 98.1%. However, when the length of the mode spot converter is less than 200 μm, it can be found that the more total steps there are, the higher the mode spot conversion efficiency is. Additionally, it can be observed that for the two linear envelope types, $y=1-\frac{x}{L}$ and $y={\left(\frac{x}{L}\right)}^{2}$, when the number of steps is 7, the mode spot conversion efficiency can exceed 90% within a certain range of mode spot converter lengths.

By analyzing the variation in the mode spot conversion efficiency with L for the different linear envelopes depicted in Figure 6, it can be observed that during the initial stages of either rising or falling steps, a gradual change is required. Specifically, a smaller rate of change corresponds to a higher mode spot conversion efficiency, which aligns with the envelope profile of $y=(\cos\frac{\pi x}{L}-1)/2$. The linear envelope $y=\sqrt{\left(\frac{x}{L}\right)}$, it exhibits a slower change at the beginning of the descent and a faster decrease later on. This type of linear mode spot converter achieves its maximum conversion efficiency with a relatively short coupling distance. Conversely, the mode spot converter with a $y={\left(\frac{x}{L}\right)}^{2}$ linear envelope behaves oppositely. In summary, when light gradually merges into the waveguide or gradually escapes from the inner waveguide, a slow variation in step height is necessary to achieve a high mode of spot conversion efficiency. The envelope steps of the profile $y=(\cos\frac{\pi x}{L}-1)/2$ satisfy such a pattern. From the coupling of the optical fiber into the mode spot converter and its transmission into the LNOI waveguide throughout the entire process, the overall transmission efficiency, including the mode matching efficiency between the step-structured mode spot converter and the optical fiber, can be calculated to be 95.35%.

## 4. Conclusions

In conclusion, we analyzed the coupling process between a stepped SSC and an optical fiber. By using FDE and FDTD simulations, a coupling structure for a broadband, high-efficiency fiber-to-chip SSC is designed. Specifically, a $y=(\cos\frac{\pi x}{L}-1)/2$ linear mode spot converter is designed that can be efficiently coupled with a conventional single-mode fiber at a mode-field diameter of 9.8 μm, with a coupling efficiency of up to 95.35%. Finally, based on the variation patterns of the curves, this paper analyzes the impact of different step-structured linear envelopes on the optical transmission efficiency and explains how the linear envelope affects the conversion efficiency of the step-structured mode spot converter. This lays the foundation for further realizing low-loss coupling structures.

## Figures and Tables

**Table 1:** Physical quantities used in the mathematical model.

Number	Physical Quantities	Expression
1	Refractive indices	*n*
2	Reflectivity	*R*
3	Transmittance	*T*
4	Overlap integral factor	*η*
5	Electric field strength	*E*
6	Coupling coefficient	*a*
7	Infinitesimal area element	*dA*

## Data Availability

The original contributions presented in this study are included in the article, and further inquiries can be directed to the corresponding author.
